# A Token Classification-Based Attention Model for Extracting Multiple Emotion–Cause Pairs in Conversations

**DOI:** 10.3390/s23062983

**Published:** 2023-03-09

**Authors:** Soyeop Yoo, Okran Jeong

**Affiliations:** School of Computing, Gachon University, 1342 Seongnam-daero, Seongnam 13120, Republic of Korea; bbusso@gachon.ac.kr

**Keywords:** emotion–cause pair extraction, emotion–cause extraction, conversational AI, token classification, pre-trained language model

## Abstract

People exchange emotions through conversations with others and provide different answers depending on the reasons for their emotions. During a conversation, it is important to find not only such emotions but also their cause. Emotion–cause pair extraction (ECPE) is a task used to determine emotions and their causes in a single pair within a text, and various studies have been conducted to accomplish ECPE tasks. However, existing studies have limitations in that some models conduct the task in two or more steps, whereas others extract only one emotion–cause pair for a given text. We propose a novel methodology for extracting multiple emotion–cause pairs simultaneously from a given conversation with a single model. Our proposed model is a token-classification-based emotion–cause pair extraction model, which applies the BIO (beginning–inside–outside) tagging scheme to efficiently extract multiple emotion–cause pairs in conversations. The proposed model showed the best performance on the RECCON benchmark dataset in comparative experiments with existing studies and was experimentally verified to efficiently extract multiple emotion–cause pairs in conversations.

## 1. Introduction

People express their emotions through various methods and communicate with others based on such emotions. Recent research has focused on human emotions based on numerous datasets, including text and image datasets. In particular, tasks that classify or extract emotions based on text data are being actively studied in the field of natural language processing. Similar to many other tasks in this field, various studies are being conducted through the development of pre-trained language models, such as BERT [1] and ELMo [2], and most models show a high accuracy or outstanding performance on the benchmark dataset [3,4,5,6].

Studies on emotions in natural language processing are primarily focused on classification tasks. In the past, the primary focus was on tasks that classify human emotions as positive or negative in a given text. Some recent studies have classified various types of emotions by diversifying their classification. In addition, the domain of studies is also expanding from one-way text data, such as news and novels, into two-way dialogue data, which mainly reflects human emotions [7,8,9,10,11].

Because communication occurs in both directions, human emotions are reflected in various ways. Conversations usually show multiple emotions in each utterance, e.g., sad emotions changing into happy emotions through conversations rather than continuing as a single emotion. In addition, the causes of emotions can appear in various conversations. Through conversation, a person can understand another person’s feelings and respond according to the cause of the emotion. For example, if someone says, “I messed up on my mid-term exam so badly today,” a listener can identify that the cause of the speaker’s sadness from the utterance is the exam, and reply with, “It’s just one exam. You’ll get better results next time.” Because chatbots that generate conversations should be able to determine not only the emotions of a speaker but also their causes, research is being conducted to extract emotions and their causes as a single pair in a conversation [12,13,14,15,16].

Extracting an emotion and its cause in a single pair is called an emotion–cause pair extraction (ECPE) task, for which various models have been proposed, albeit with several limitations. First, most models are inefficiently constructed. Many studies [17,18,19,20,21,22,23] have proposed end-to-end models that take several steps to pair emotions and their causes. Emotions are extracted using an emotion extraction model, and the cause is extracted using an extraction model. The candidates for emotions and their causes are generated into pairs through a pairing process. These methods can improve the overall performance by improving the performance of each model. However, an inefficiency problem occurs in that the performance of one model can eventually affect that of the next model. Additionally, there is a problem of ignoring the mutual dependency between emotions and causes.

Second, when emotion–cause pair extraction is applied with a single model, only one emotion–cause pair can be extracted. To solve the first limitation, although some singular models extract emotion–cause pairs, such as those using a deep learning approach, a single model can usually extract only one emotion–cause pair. However, because a conversation is complicated by various emotions and their causes, it must be possible to find multiple emotion–cause pairs concurrently.

Since emotions and causes interact with each other in a conversation and influence the next conversation, it is very important to figure out emotions and causes efficiently while solving existing limitations. To solve these limitations, we propose a one-stage token classification model for emotion–cause pair extraction during a conversation. We suggest a new problem-solving methodology that differs from existing studies in that it extracts multiple cause-emotion pairs simultaneously. We also propose and implement a model that utilizes a pre-trained language model.

Our proposed model has the following contributions:

- **BIO (beginning–inside–outside) tagging scheme:** We propose a novel methodology used to apply the BIO tagging scheme to extract an emotion–cause pair from a conversation. Many models define and solve problems through a question/answering (QA) method, e.g., a machine reading comprehension (MRC) task, which reformulates conversations into questions to find an emotion–cause pair and designs problems used to find answers to such questions. To solve a problem in this manner, separate preprocessing is required to formulate a conversation into a question. The proposed method does not require this process because it allows the use of a given conversation as is and solves the limitations of existing models.
- **Multiple emotion–cause pairs:** Multiple emotion–cause pairs can be extracted from a single sentence. Only one emotion–cause pair can typically be extracted from a given conversation by a single model. However, our proposed model allows individual tagging for each token in a sentence by applying the BIO tagging scheme, and thus we can extract multiple emotion–cause pairs from a given conversation. In other words, it can be used in real-world environments because it is possible to find different emotions and their causes in a conversation.
- **One-stage token classification-based attention model:** We propose an efficient model for emotion–cause pair extraction during a conversation. Most existing models apply emotion and cause extraction separately, and then formulate pairs through a step-by-step approach. These models are inefficient because the performance of each step also affects the performance of the next step, and multiple steps are required. We propose an efficient method of extracting multiple emotion–cause pairs while converting all the steps into a single model, thereby reducing the cost required to generate responses when later applied to chatbots.

In this study, we introduce the proposed model in detail, and to verify its strength, describe comparative experiments conducted with existing studies. Section 2 presents existing studies, describes their limitations, and explains how the proposed model solves such limitations. Section 3 describes the proposed model in detail. Section 4 presents the experimental environment and experimental results and summarizes the results. Finally, Section 5 summarizes the conclusions of the study and presents areas of future research.

## 2. Related Work

### 2.1. ECE and ECPE

Emotion–cause extraction (ECE) tasks aim to identify the cause of emotion in a text when an emotion is given. Early ECE studies mainly used rule-based methods [24,25]. The authors of [12] first published an annotated dataset for ECE tasks. Research is being conducted to determine the cause of a given emotion using rule-based methods, and various studies using classical machine learning and deep learning-based models have been conducted [26,27,28,29]. The main limitation of ECE tasks is that the mutual dependency between the cause and the emotion might be ignored. This is because the emotion must be annotated first, and then the cause can be extracted.

The authors of [13] first proposed the emotion–cause pair extraction (ECPE) task, which, unlike the ECE task, does not provide emotions. To solve this problem, ECPE extracts all potential pairs of emotions and their causes from a text because cause extraction is highly dependent on emotion annotation. Unlike the ECE task, this is considered a relatively tricky task because emotions and their causes must both be extracted.

Studies on ECPE [15,17,18,19,20,21,22,23] have solved this problem by dividing it into emotion and cause extraction stages to extract emotions and their causes. When finding the emotion–cause pair in a two-step process, the emotion is extracted in the first step, the cause is extracted in the next step, and the method of connecting the extracted cause and the result of the emotion differs according to the specific study. Finally, an emotion–cause pair is created using techniques such as a 2D matrix or rank [17,18,30]. Models based on neural networks, such as TSAM [31], have also been studied, along with various methods for achieving ECPE.

The ECPE task is a proposed task to address the neglect of the mutual dependency between emotion and cause, which is a major limitation of the ECE task. Many studies improve performance by applying deep learning models, but most of them still apply a two-step process. This still has the problem of the mutual dependency between emotion and cause because the two-step process-based methods extract emotions and causes in individual processes. We propose a model to extract multiple emotion–cause pairs at once using a token classification-based attention model to address this problem.

### 2.2. RECCON

Recognizing an emotion cause in conversations (RECCON) is a new benchmark proposed in [14] and is a dataset containing more than 1000 conversations and emotion–cause pairs for more than 10,000 utterances. Unlike traditional ECE and ECPE, which are mainly based on documents such as news and novels, RECCON is based on the DailyDialog [32] and IEMOCAP [33] datasets, which are emotion-based conversational datasets.

**Figure 1:**
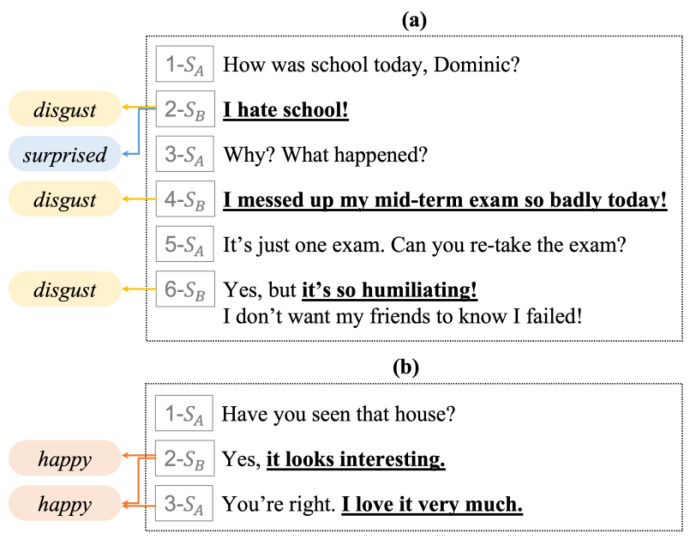
Examples of RECCON dataset; (**a**) example of the utterance having different emotions, and (**b**) example of two or mor causes representing a single emotion.

Figure 1 shows examples of the RECCON dataset. In this dataset, the utterances of the n-turns exchanged by speakers A and B comprise one dialogue. For each utterance in a single conversation, emotions are classified into seven categories: happiness, disgust, surprise, sadness, anger, fear, and neutrality, and are annotated by determining the reason behind each emotion. Here, $S_{A}$ and $S_{B}$ represent speakers A and B, respectively, and the numbers displayed in front of them represent their turn in the conversation. The emotions shown on the left side show the emotions corresponding to each turn. The clauses in italics and bold indicate the cause of the emotion, and they refer to the emotion due to the corresponding causes using an arrow.

As in example Figure 1a, different emotions can be created depending on the utterance being targeted, and the same reason can be the cause of other emotions. “I hate school!” in the second turn may be the cause of speaker B’s feeling of disgust, and simultaneously, speaker B’s surprised feeling in the third turn. As in this example, finding emotion–cause pairs in a conversation is challenging because multiple emotions can appear, and one cause can also lead to multiple emotions.

In addition, two or more causes may represent a single emotion, such as the utterance corresponding to the third turn in Figure 1b. Speaker A feels happy in the third turn based on two clauses: speaker B’s statement that “it looks interesting” in the second turn and speaker A stating “I love it very much” in the third turn. Because there may be more than two causes for expressing one emotion, the model should consider this when extracting emotion–cause pairs.

We leveraged the dataset provided by the RECCON benchmark. Existing datasets for ECPE tasks are mainly in Chinese or are extremely sparse. However, RECCON is used for model learning and experimentation because, in this dataset, both the emotion and the cause are annotated for English conversation datasets. As shown in the examples in Figure 1, only one emotion–cause pair must not be extracted from the conversation dataset. Contrarily, multiple emotion–cause pairs must be extracted for a given target utterance.

Unlike previous studies, our proposed model presents a new problem-solving method for solving these problems and applies a one-stage approach. Through the newly proposed model, multiple emotion–cause pairs can be extracted simultaneously to solve the limitations of existing models.

## 3. Proposed Methodology

We propose a one-stage token classification model for extracting emotion–cause pairs from a conversation. Unlike previous studies, the proposed methodology solves the problem using token classification to extract the emotion–cause pairs after only a single step. This section describes the proposed model in detail.

### 3.1. Task Definition

We propose training the model in a novel way to extract multiple emotion–cause pairs from a given conversation using a single model. Existing studies have proposed a multi-step end-to-end model for emotion–cause pair extraction or using a question/answering method to solve problems with a single model. These methods are relatively inefficient because the end-to-end model requires multiple steps, and solving problems in the question/answering method requires generating questions to determine answers to a given conversation.

Unlike previous studies, we solved this problem by applying the BIO tagging scheme to efficiently extract multiple emotion–cause pairs. The proposed model tokenizes all given conversations and then classifies the tokens that cause each token into the corresponding emotion tag. Because each token can be classified into an emotion tag, multiple emotion–cause pairs can be extracted simultaneously with a single model.

The task that we want to solve is defined as follows: Given the dialogue history $D\left(u_{t}\right)=\left(u_{1},u_{2},\cdots ,u_{t}\right)$ and the target utterance $u_{t}$, all emotion–cause pairs $ECP\left(u_{t}\right)=\left\{\left(e_{1},c_{1}\right),\left(e_{2},c_{2}\right),\cdots ,\left(e_{m},c_{n}\right)\right\}$ are extracted for the target utility. Here, $e_{m}$ represents an emotion and $c_{n}$ represents the result of finding the cause of the emotion. We used $D\left(u_{t}\right)$ and $u_{t}$ as inputs to solve the problem with a one-stage model.

### 3.2. Representation Using BIO Tags

According to the defined problem, we must determine one or more emotion–cause pairs using a given dialogue history and the target interference. We used the BIO tagging scheme to solve this problem. The BIO tagging method is also called an IOB format. A description of the tags used for the model is provided in Table 1. The B-prefix refers to the beginning and represents the token for which the cause of the emotion begins in an utterance. The I-prefix refers to a token inside and represents a token corresponding to the cause of the emotion in an utterance. Finally, O represents a token that does not belong to any emotion and does not correspond to a cause.

**Figure 2:**
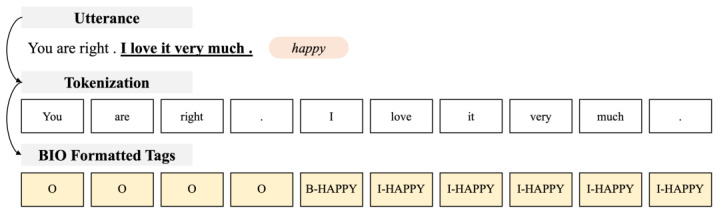
Example of the proposed BIO formatted tags.

Figure 2 shows an example application of the BIO format. It is assumed that the utterance “You are right. I love it very much” occurs. Tokenization is used based on the space. In the cause “I love it very much,” the initial token “I” is represented as [B-HAPPY], and the remaining tokens are expressed as [I-HAPPY]. The utterance “You are right,” which does not belong to any emotion, is described as [O]. This example is intended to easily represent how we apply it and should only be referred to as an understanding of the application method because it differs from the actual tokenization result.

### 3.3. Token Classification-Based Attention Model

We propose a token classification-based emotion–cause pair extraction model used in conversations and aim to simultaneously extract human emotions and causes from a given conversation. To learn the proposed model, it is applied by changing the given conversation into a form that enables learning. In particular, to solve the problem, we need to preprocess existing datasets by applying the token classification method for extracting the emotions and causes simultaneously.

**Figure 3:**
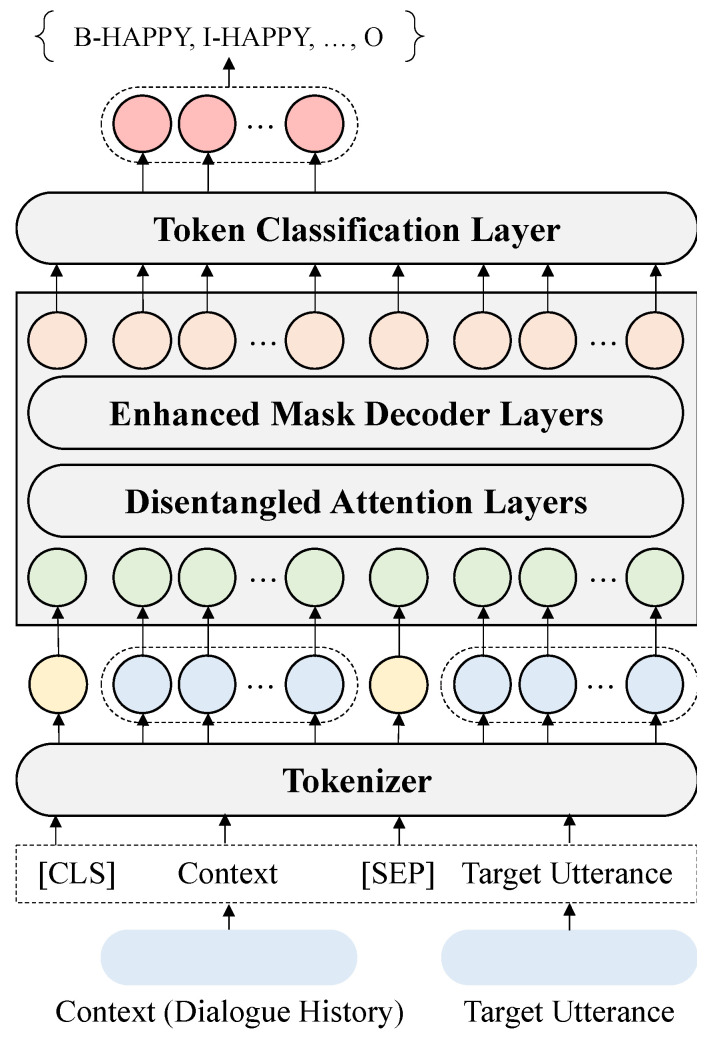
Model architecture.

Figure 3 shows the structure of the proposed model. The dialogue history (context) and target utterances are input into the model. When going through the tokenization process, the [CLS] token is added to the front of the dialogue history, i.e., the context, and the [SEP] token is added between the context and the target utterance to distinguish between them. We enter the tokenized result as “[CLS] + tokens for context + [SEP] + tokens for target utterance” into the pre-trained language model. Based on experimental results, the proposed model uses Microsoft’s DeBERTa-large model [34].

Since the disentangled attention model proposed by the DeBERTa [34] model is used in our proposed model, the cross-attention score between tokens i and j of the disentangled self-attention can be expressed as Equation (1) below. i and j indicate the position within the sequence.

(1) $$A_{i,j}=H_{i}H_{j}^{⊺}+H_{i}P_{j|i}^{⊺}+P_{i|j}H_{j}^{⊺}$$

In Equation (1), $H_{i}$ represents the token vector at position i and $P_{i|j}$ represents the relative position at position i from the token at position j. As proposed in DeBERTa [34], the attention weight is computed using the cross-attention score of the token and relative position embedding.

Subsequently, the token classification layer finds the classification result corresponding to each token. The proposed model shows the results for a BIO-formed tag. Based on the BIO-formed tag for each token, we finally determine the emotion and cause pairs and make them the final results. First, we find the “B-” prefix in the token result, and then determine the location of the tokens with the “I-” prefix in the original utterance until the “O” tag is output. The tag after the “B-” prefix becomes the emotion, and the sentence combining each token becomes its cause.

The model we propose can extract the emotion–cause pairs of the target utterance only by entering conversations as input. In addition, because BIO-formed tags and token classification are utilized, our model can extract multiple emotion–cause pairs for one input. For example, given the sentence “I feel good because the weather is so nice, but I get angry at myself when I think about failing the test!”, our model can extract {HAPPY, “the weather is nice”} and {ANGRY, “failing the test”} at once. In other words, our proposed model is relatively efficient because it can extract multiple emotion–cause pairs using a single model, unlike existing methods, and actually considers the mutual dependency between emotions and causes.

## 4. Experiments

### 4.1. Dataset and Evaluation Metrics

We utilized the datasets provided by the RECCON [14] benchmark for the experiments. The RECCON dataset contains more than 1000 conversations and emotion–cause pairs for more than 10,000 utterances. In particular, we used the RECCON-DD dataset using DailyDialog [32] as the original data. Table 2 shows the statistics for the RECCON-DD dataset. The RECCON-DD dataset is classified into seven emotions: happiness, surprise, anger, sadness, disorder, and fear. It is divided into 27,915 for the training set, 1185 for the valid set, and 7224 for the test set.

The existing RECCON-DD dataset is configured to solve causal span extraction, a subtask defined in the RECCON benchmark, such as an MRC task. When context and a question are given, it is in the form of finding an answer. Context serves as a type of conversation history by combining utterances in the conversation of the existing DailyDialog dataset. The question is built using a template to provide target utterances, evidence utterances, and emotions as a single question. This template consists of the following forms: {*The target utterance is [target utterance]; The evidence utterance is [evidence utterance]; What is the causal span from the context that is relevant to the target utterance’s emotion [emotion] ?*}. The answer is annotated as the cause of the emotion.

Although it would be beneficial to take advantage of the existing dataset, to extract the emotion–cause pair, we proceeded with a preprocessing of the dataset to solve it in a novel manner, rather than applying the MRC method. Using the template format of the question, the target utterance, evidence utterance, and emotion are all extracted and organized into columns.

The evaluation metric also utilizes the metric used in the RECCON benchmark for performance verification. The RECCON benchmarks employ experimental methods utilized in question-answering (QA) tasks because they solve existing conversation datasets in the form of QA tasks. The F1 score is also calculated for a negative sample that cannot be answered. Here, Pos. F1 is the F1 score for a positive sample. The F1 score for a negative sample is expressed as Neg. F1. The final average of the two F1 scores is expressed as macro F1 and is used to evaluate the final results of the experiment.

### 4.2. Implementation Details

The experiment was conducted in the Google Colab environment. We used a Tesla T4 with 16 GB of memory, which is one of the GPUs provided by Google Colab, and implemented and experimented on the models using PyTorch. We applied Microsoft’s DeBERTa-large [34] model for model learning, which is published on Huggingface. We set max_seq_len to 512, the batch size to 4, and the learning rate to $3\times 10^{-5}$. We applied the optimization using the Adam optimizer with a weight decay of 0.01, and the epoch was set to 3 during training. For the training, we leveraged the RECCON-DD dataset provided by the RECCON benchmark, and applied the same methods used by the benchmark for the evaluation.

### 4.3. Results

We conducted comparative experiments with existing studies to verify the performance of the proposed model. For the comparison, models that applied an emotion–cause extraction task on the RECCON-DD dataset were set up and compared with existing studies. We experimented on the recently released RankCP [17], ECPE-MLL [30], ECPE-2D [18], MuTE-CCEE [35], DAM [36], KBCIN [37], TSAM [31], and KEC [38] models released and experimented on the RECCON benchmark, and the RoBERTa-Base and RoBERTa-Large model [14], which became the baseline models for the benchmark.

RankCP [17] applies an end-to-end emotion–cause pair extraction method that ranks the utterance pairs first and then applies a one-stage neural approach. The ECPE-MLL [30] model uses a joint multilabel scheme, which is a framework consisting of a module for extracting emotion and two modules for extracting the cause of the emotion. The ECPE-2D [18] model applies an end-to-end method using a 2D transformer network for emotion–cause pair extraction. The MuTE-CCEE [35] is an end-to-end multi-task learning framework for extracting emotions, emotion cause, and entailment in conversations. 

The DAM [36] model proposes a discourse-aware model by using a multi-task learning framework and a gated graph neural network. KBCIN [37] proposes a knowledge-bridged causal interaction network with commonsense knowledge as three bridges: semantics-level, emotion-level, and action-level. The TSAM [31] model utilizes a two-stream attention approach using three modules: an emotion attention network, a speaker attention network, and an interaction module. The KEC [38] model uses social commonsense knowledge to build a knowledge-enhanced conversation graph and achieve the state-of-the-art (SOTA) on the RECCON benchmark before the model we propose.

Table 3 shows the comparative experimental results of the models conducting an emotion–cause extraction task on the RECCON-DD dataset. We calculate Pos. F1 and Neg. F1 for each positive and negative example, respectively, and macro-F1 is calculated to verify the performance. We conducted a comparative experiment by applying the DeBERTa-base and DeBERTa-large models. Based on these results, we propose an approach that applies the DeBERTa-large model.

Because we achieved new state-of-the-art (SOTA) results on the RECCON dataset as the final verified performance, the proposed model is meaningful in that it can extract both emotions and causes simultaneously by entering only the existing conversation history and the target utterance; that is, the most recent utterance. Despite extracting multiple emotion–cause pairs simultaneously through only a single stage, it performed relatively better than previous approaches and achieved the SOTA on the RECCON dataset.

Table 4 shows the results of a comparative experiment on the impact of emotion information on our proposed model. “Emo Accuracy” shows the accuracy of the emotion classification compared to the actual emotion label. “Gold Emotion” is the result of our proposed model including real emotion information (gold standard emotion label), and “No Emotion” is the result of only entering conversations without including emotion information and inferencing emotion and cause as a pair. The case of “Gold Emotion” showed relatively high performance on all indices because we gave the emotion label as a hint to the model. The “No Emotion” model we used for comparison with existing studies showed relatively low performance because both emotion classification and cause of emotion inference must be possible simultaneously because it did not provide an emotion label. However, we can see that our proposed model performed complex tasks well because it showed about 82% accuracy on emotion classification and did not have a large performance difference in cause inference.

## 5. Discussions

We propose a model for extracting a speaker’s emotions and their causes from a given conversation. The F1 score achieved through the comparative experiments shows a better performance than that of the other existing studies. In addition to the performance, it was confirmed through several examples that various emotions and causes, as a characteristic of the proposed model, can be extracted simultaneously.

Table 5 presents the inference results for the proposed model. Each conversation consists of utterances between speakers A and B, and “*” represents a target utterance. The results of the extracted pairing of an emotion and its cause are shown in bold when the conversation history, that is, the context and target intervention, are entered into the model.

The first example extracts are {HAPPINESS, “What a nice day!”}, {HAPPINESS, “enjoying the sunshine”}, {HAPPINESS, “sunshine and wind remind me of our honeymoon”}, from the first, second, and third turns, respectively. It can be seen that multiple emotion–cause pairs can be extracted simultaneously by simply entering a dialogue into the model.

The second example extracts are {SADNESS, “disaster”} and {DISGUST, “been covered over by mud”}. Different emotions were extracted by extracting two results with one input. The causes corresponding to each emotion were found, and the results are shown. This shows the strength of the proposed model as a possible approach because we apply the token classification method rather than the QA method as in previous studies.

The proposed model can extract emotions and causes simultaneously without any process when a conversation history is given and a target utterance is provided. The performance of the model was verified through comparative experiments with previous studies, and the availability of the model was confirmed. In particular, as an essential part of the model, emotions and causes are extracted in a single stage, and the problems are redefined and solved through token classification rather than in the form of QA tasks, and thus, it is possible to respond to multiple causes for emotions in a given context.

However, the RECCON dataset used as a learning dataset has a limitation in that the imbalance in the emotional classification is severe. Approximately 70% of all datasets were classified as having HAPPINESS emotions, and four other emotions were divided into the remaining distributions. In addition, positive emotions were classified into one HAPPINESS emotion, and emotions corresponding to negative emotions were subdivided. An expansion of the dataset is required to improve the model performance. By segmenting positive emotions and expanding the data corresponding to other emotions, the performance of the model is expected to improve.

## 6. Conclusions

In conversation, a person’s emotions and causes have an important influence on the subsequent conversation, so there are many tasks to extract emotions and causes in conversational AI. Many existing studies conduct emotion–cause pair extraction tasks through a two-step process by dividing the steps into emotion extraction and cause extraction, or into redefining and performing tasks as a type of MRC task. However, they still have a limitation of ignoring the mutual dependency between emotions and causes. We propose a token classification-based attention model that can extract multiple emotion–cause pairs to address the existing limitations. Specifically, we redefine the problem using a token classification task, which allows us to extract emotions and causes simultaneously in a single step. We verify the proposed model through comparative experiments with existing models and the proposed model achieves the SOTA on the RECCON dataset.

Because it can simultaneously extract human emotions and causes in conversation, the proposed model can be used to generate new answers based on emotions and causes in the future. Although existing models that generate answers based on emotions in conversations have been studied, such approaches have limitations in solving these problems, and the answers must also change depending on the cause of the emotions. Therefore, it is possible to improve the dialogue generation model by generating a dialogue based on the proposed model.

## Figures and Tables

**Table 1:** BIO-formatted tags.

Prefix	Explanation	Tags (Prefix + Emotion)
B-	The beginning of a token that belongs to the [emotion]	B-HAPPY B-SURPRISE B-ANGER B-SAD B-DISGUST B-FEAR
I-	A token is inside the tag	I-HAPPY I-SURPRISE I-ANGER I-SAD I-DISGUST I-FEAR
O	A token belongs to no [emotion]	O

**Table 2:** Dataset statistics.

Emotion	Train	Valid	Test
happiness	22,095	785	4520
surprise	2205	112	576
anger	1513	139	982
sadness	1269	114	806
disgust	555	10	192
fear	278	25	148
**Total**	27,915	1185	7224

**Table 3:** Overall results of emotion–cause pair extraction model.

Model	Pos. F1	Neg. F1	Macro F1
RankCP [17]	33.00	97.30	65.15
ECPE-MLL [30]	48.48	94.68	71.59
ECPE-2D [18]	55.50	94.90	75.23
RoBERTa-Base [14]	64.28	88.74	76.51
RoBERTa-Large [14]	66.23	87.89	77.06
MuTE-CCEE [35]	69.20	85.90	77.55
DAM [36]	67.91	89.55	78.73
KBCIN [37]	68.59	89.65	79.12
TSAM [31]	70.00	90.48	80.24
KEC [38]	66.76	95.74	81.25
DeBERTa-base	68.56	94.41	81.48
**Ours**	**71.27**	**96.13**	**83.70**

**Table 4:** Comparison results for the impact of emotion information on our proposed model.

Emotion	Emo Accuracy	Pos. F1	Neg. F1	Macro F1
Gold Emotion	-	76.25	96.52	86.39
No Emotion	82.03	71.27	96.13	83.70

**Table 5:** Inference results of our proposed model.

No.	Inference results
1	A: **What a nice day! *(HAPPINESS)*** B: Yes. How about going out and **enjoying the sunshine** on the grass? ***(HAPPINESS)*** A: Great, let’s go! B: Hey, darling, I think I might have a little heatstroke from being in the sun all day. I was so relaxed. It felt as if I were in another world. A *: Exactly. You know, the **sunshine and wind remind me of our honeymoon.** You remember? The island, the sound of the waves, the salty sea air and the sunshine... ***(HAPPINESS)***
2	A: Well, Yuri, tell me about it. B: I’m sorry I can’t bring better news, sir. The site is a **disaster. *(SADNESS)*** A: That’s what I was afraid of. B: It is not only the earthquake, sir. But the mudslides. Much of the **north half of the site has been covered over by mud**. ***(DISGUST)*** A: Mud? But Ivan told me there were no mudslides in that district. I thought all the mudslides were down in Chichitango. B: That’s what we thought, sir. That’s what the news reported. But there was one little mudslide in our district too. Right above our site. A *: Oh, that’s terrible! What bad luck! I wish we had never come to this country. But, if it isn’t the strikes and the revolutionaries, it’s the earthquakes. Our operations here are finished!

* denotes the target utterance.

## Data Availability

Not applicable.
